# Cohort Studies on Chronic Non-communicable Diseases Treated With Traditional Chinese Medicine: A Bibliometric Analysis

**DOI:** 10.3389/fphar.2021.639860

**Published:** 2021-03-19

**Authors:** Yiwen Li, Yanfei Liu, Jing Cui, Hui Zhao, Yue Liu, Luqi Huang

**Affiliations:** ^1^ Cardiovascular Center of Xiyuan Hospital, China Academy of Chinese Medical Sciences, Beijing, China; ^2^ China Center for Evidence-based Medicine of TCM, China Academy of Chinese Medical Sciences, Beijing, China; ^3^ National Clinical Research Center for TCM Cardiology, Beijing, China; ^4^ Institute of Clinical Pharmacology of Xiyuan Hospital, China Academy of Chinese Medical Sciences, Beijing, China

**Keywords:** cohort study, chinese medicine, bibliometric analysis, non-communicable diseases, evidence-based medicine

## Abstract

Cohort studies investigating the treatment of chronic non-communicable diseases (NCDs) with traditional Chinese medicine (TCM) have considerably accumulated in recent years. To systematically and for the first time present the achievements and dilemmas of cohort studies, strict inclusion and exclusion criteria were used to search publications from the Web of Science, PubMed, Embase, Cochrane Library, and China National Knowledge Infrastructure databases for cohort studies on NCDs with TCM since the establishment of these databases. Information on the year of publication, exposure factors, diseases, and outcome indicators was obtained, and a literature quality assessment and bibliometric descriptive analysis were conducted. A total of 182 published articles involving 1,615,106 cases were included. There were 110 non-prospective cohort studies and 72 prospective cohort studies. The diseases involved in the cohort studies were, in the order of the number of published articles, malignant tumors (82 articles, 45.05%), cardiovascular diseases (35 articles, 19.23%), neurological diseases (29 articles, 15.93%), chronic kidney diseases (16 articles, 8.79%), liver cirrhosis (8 articles, 4.40%), diabetes mellitus (8 articles, 4.40%), and chronic respiratory diseases (4 articles, 2.20%). The study participants were mainly from China (177 articles, 97.25%). The number of cohort studies increased significantly in the last 5 years (65 articles, 35.71%), and following the Newcastle-Ottawa Scale (NOS) literature quality evaluation, the number of articles that received a score of four to five was high (116 articles, 63.73%), and the overall quality needs to be improved. The application of cohort studies in the field of TCM for the prevention and treatment of NCDs has developed rapidly in the past 5 years, focusing on the prevention and treatment of tumors as well as cardiovascular and cerebrovascular diseases. However, the design and implementation of cohort studies still have considerable limitations. To provide more clinical evidence, researcher should actively cooperate with evidence-based methodologists and standardize the implementation of cohort studies.

## Introduction

Chronic non-communicable diseases are a group of chronic conditions other than acute infections or parasitic injuries, maternal and perinatal conditions, or nutritional deficiencies (Wagner and Brath, 2012). With global economic development and modernization, the economic burden has shifted from communicable diseases to NCDs (Omran, 1971). NCDs are the leading cause of death globally, accounting for 71% (41 million) of the 57 million deaths worldwide in 2016, and the number is expected to increase further (World Health Organization, 2018a). Cancer, cardiovascular diseases, diabetes, and chronic respiratory diseases account for the highest morbidity, mortality, and disability rates (World Health Organization, 2018b). Such a huge impact on individuals and the society has hastened the need for the prevention and control of NCDs with multiple therapies.

As complementary and alternative medicine is widely used in Asia and has a global impact, the role of traditional Chinese medicine (TCM) in the prevention and treatment of chronic NCDs has received increasing attention worldwide, and approximately 61% of anticancer drugs and 50% of cardiovascular drugs were developed from natural herbal products in recent years (Newman and Cragg, 2020; Zhao et al., 2020), especially since the Nobel Prize was awarded for research on artemisinin from TCM (Ma et al., 2020). Natural products and natural product-derived compounds have proven to be effective and to have a mechanism against NCDs. The interaction and overlap of pathways in different diseases had made the use of the same TCM treatment for different diseases possible (Huang et al., 2018; Zhang and Wei, 2020). Since the outbreak of the coronavirus 2019 (COVID-19) epidemic that swept across the world in early 2020, integrated medicine therapies have played a tremendous role in the rapid control of this epidemic in China (Hu et al., 2020). In recent years, the effect of TCM on preventing and treating chronic NCDs and improving the clinical prognosis of some diseases has been confirmed by an increasing number of clinical studies (Hershman et al., 2018; Yang et al., 2012).

Cohort studies are suitable for obtaining outcomes that are closest to real-world TCM practice, with characteristics such as a long treatment cycle and an individualized treatment plan (Liu, 2007). In recent years, the number of cohort studies in the field of TCM has shown a significantly growing trend, involving many diseases especially NCDs. The clinical practice and effects of TCM have been emphasized in cohort studies, although the quality of research needs to be improved. Cohort studies in the field of TCM are mostly interventional cohort studies, i.e., with TCM (including TCM formulas, acupuncture, etc.) treatment as the exposure. A specific population is divided into exposure and non-exposure groups, and the differences in the incidence of the endpoint events between the groups are tracked and observed for a period of time, in order to further evaluate the clinical efficacy of the prevention and treatment of major diseases (Zhang et al., 2019). No randomization is used in these studies, and the willingness of subjects to receive treatment is monitored, with an increased subject compliance, a long follow-up period, and favorable long-term endpoint indicators.

This paper considers the prevention and treatment of chronic NCDs as a research entry point, summarizes and conducts a bibliometric analysis on the current status of cohort research in the field of TCM over the past 2 decades, and discusses the scope for future research.

## Materials and Methods

### Retrieval Strategy

The Web of Science, PubMed, Embase, Cochrane library, and China National Knowledge Infrastructure (CNKI) databases were searched on a computer for literature from the time the databases began to include journal articles to October 1, 2020. The English search terms were Chinese medicine, acupuncture, herb, herbs, alternative therapy, complementary therapy, and cohort study. The Chinese search terms were *zhen jiu* (acupuncture), *zhen ci* (needle puncture), *dian zhen* (electroacupuncture), *ai jiu* (moxibustion), *tui na* (manipulation), *zhong yi* (TCM), *zhong yao* (traditional Chinese materia medica), *zhong xi yi jie he* (integrated traditional Chinese and Western medicine), *zhong cheng yao* (traditional Chinese patent medicine), *tang* (decoction), *fang* (formula), and *dui lie yan jiu* (cohort study). The retrieval formula was appropriately adapted to different databases.

### Inclusion Criteria

1) The study design was a cohort study; 2) The study had a clear classification of the exposure and non-exposure groups; 3) The exposure factors of the study were related to TCM interventions, which were defined as various dosage forms of TCM formulas, acupuncture, manipulation, etc.; 4) The study endpoints should be related to important NCDs; NCDs included in this study included malignant tumors, cardiovascular diseases (coronary artery disease, atherosclerotic heart disease, heart failure, hypertension), respiratory diseases (chronic obstructive pulmonary disease, asthma), diabetes and related complications, liver cirrhosis, chronic kidney disease (diabetic nephropathy, chronic renal insufficiency), and neurological diseases (stroke, Alzheimer’s disease, dementia); and 5) The language is limited to Chinese or English.

### Exclusion Criteria

1. Duplicate publications or articles with only an abstract and no access to the full text;
2. articles claiming to be cohort studies but were found to be randomized controlled studies, case-control studies, or cross-sectional studies after reading the full text.

### Data Extraction

Literature management was performed using the EndnoteX9 software. Data were extracted using a pre-defined data collection form and entered using epidata 3.1 software.

Information extracted included the year of publication, language of publication, region in which the study was conducted, type of cohort study reported in the article (including prospective, retrospective, and ambispective cohort studies), sample size, exposure factors for the observation groups, disease-states studies (classified according to the 11th revision of the International Statistical Classification of Diseases and Related Health Problems, ICD-11 (Lancet, 2019)), and observation indicators.

The methodological quality of each cohort study was evaluated according to the Newcastle-Ottawa Scale (Stang, 2010) item-by-item, and a total score was calculated, which was proportional to the quality of the study. The NOS scoring was done by two evaluators separately. Disagreements were resolved by further reference to the original text and by consensus, and if not, a qualified third person was asked to evaluate the study.

### Data Statistics

This study used EXCEL 2007 (Microsoft Corporation, Redmond, WA, United States) to classify, descriptively analyze, and report the data extracted from the literature. Edrawsoft 9.4 was used to draw the figures.

## Results

### General Information of the Included Articles

**FIGURE 1:**
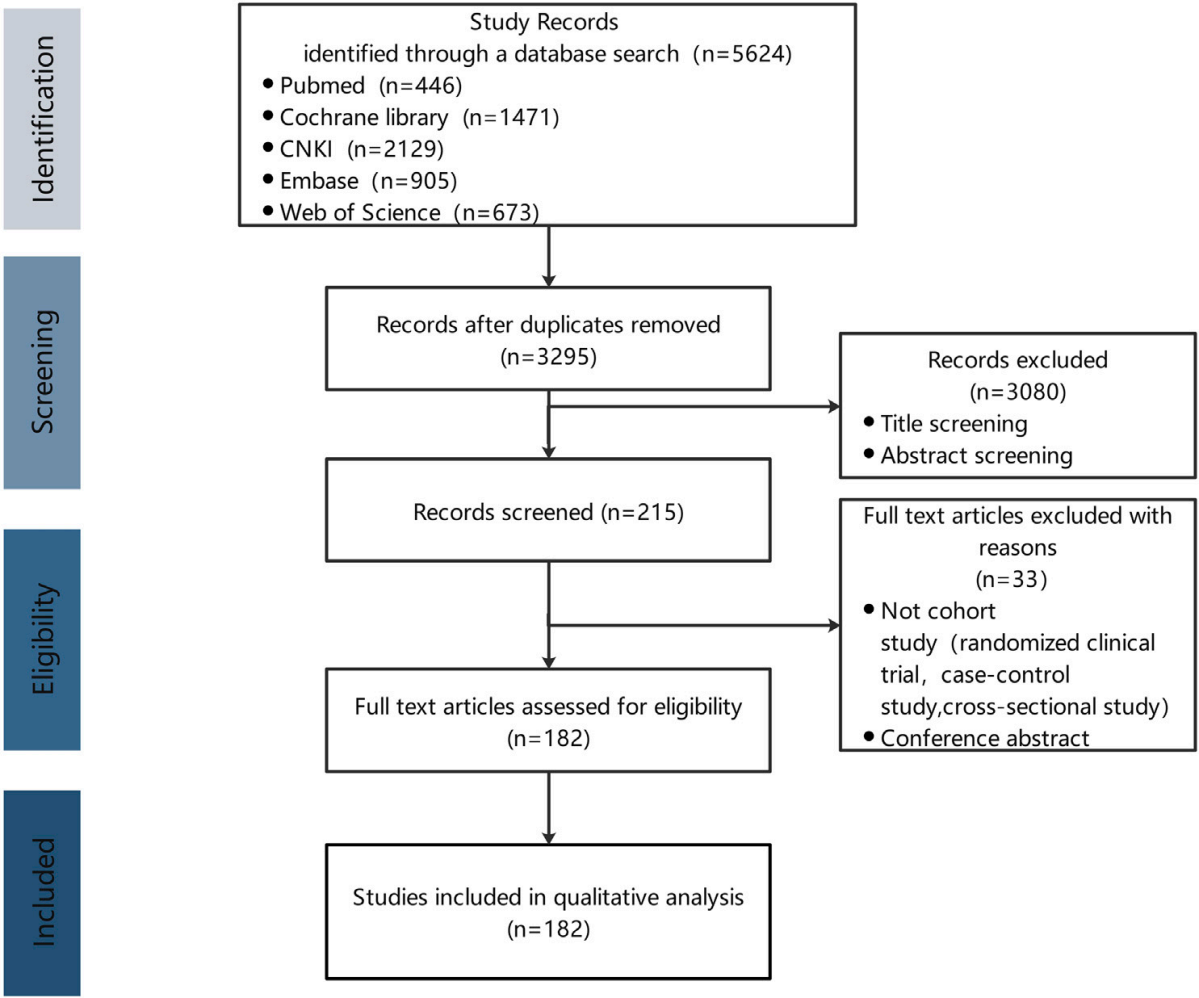
Flowchart of the literature search and study selection.

A total of 5,624 articles were retrieved and screened according to the inclusion and exclusion criteria, and 182 articles were finally included in this study for analysis (Figure 1).

A total of 89 English articles (48.90%) and 93 Chinese articles (51.10%) were included. There were 72 prospective (39.56%), 91 retrospective studies (50%), 4 ambispective (2.20%) cohort studies, and 15 inadequate trial reports (8.24%). From the 182 articles, a total of 1,615,106 cases were included in this study. Among the 21 articles (11.54%) with the number of cases >10,000, one was from South Korea, while all the others were from Taiwan. There were 25 articles (13.74%) with 10,000 ≥ number of cases >1,000, 22 articles (12.08%) with 1,000 ≥ number of cases >500, 81 articles (44.51%) with 500 ≥ number of cases >100, and 33 articles (18.13%) with 100 cases ≥ number of cases >0.

**FIGURE 2:**
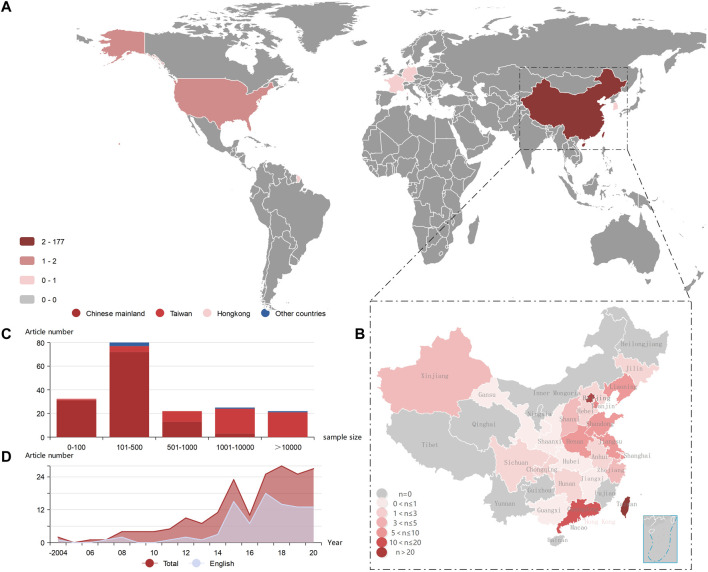
General situation of NCDs in TCM literature **(A)** Heat map of the regions where cohort studies were conducted (global). Based on the regions where the studies were conducted, the number of cohort studies conducted is proportional to the color of the region **(B)** Heat map of the regions where cohort studies were conducted (China). The number of cohort studies conducted is proportional to the color of the region. The number of cohort studies is high in Taiwan, Beijing, Shanghai, and Guangzhou **(C)** The relationship between the number of cases and the number of published articles. Red series represents China (including Chinese mainland, Taiwan, HongKong), and blue color represents other countries. **(D)** The relationship between the year of publication and number of published articles. The first two digits of the year along the horizontal coordinate have been omitted owing to space limitation. The total red area is the total number of published articles, the gray area is the number of English published articles, and the area not shaded by gray color is the number of Chinese published articles. NCD, non-communicable disease; TCM, traditional Chinese medicine.

The implementation regions included Asia, Europe, and North America. There were 177 articles from China (119 from Chinese Mainland, two from Hong Kong, and 56 from Taiwan), and 5 articles were from other countries (1 from South Korea, 2 from the United States, 1 from Germany, and 1 from France). Few articles were published before 2015 and were mainly published in Chinese journals; since 2015, the number of published articles has surged, and the proportion of English articles has increased (Figure 2).

### Exposure and Endpoints in the Included Articles

**FIGURE 3:**
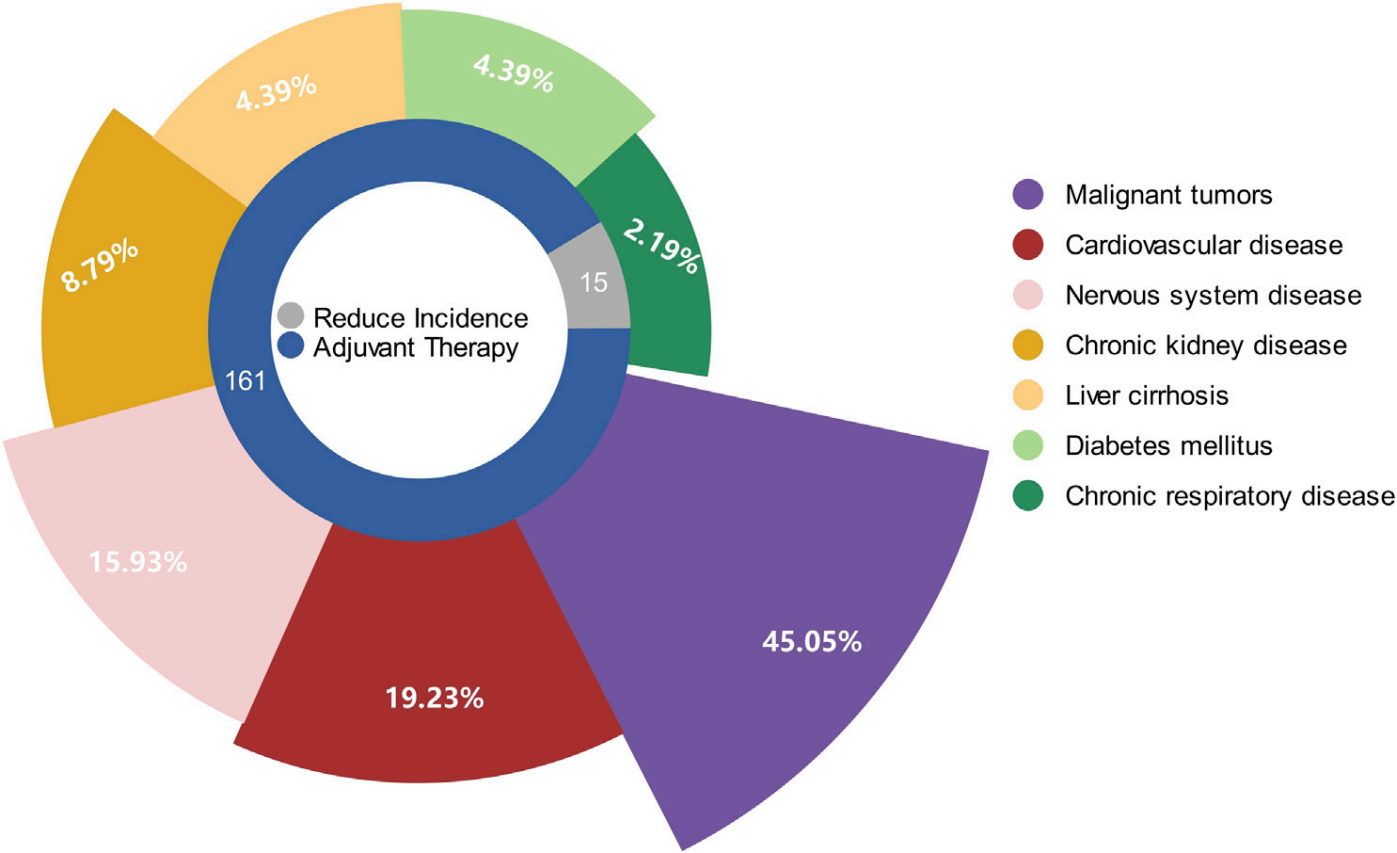
Pie chart of the disease profile in the cohort studies. The inner circle of the pie chart shows the number of articles on TCM on the prevention and adjunctive treatment of NCDs. The outer circle of the pie chart shows the number of articles on each type of disease. NCD, non-communicable disease; TCM, traditional Chinese medicine.

The diseases involved include malignant tumors (lung cancer, gastric cancer, colorectal cancer, liver cancer, breast cancer, gynecological cancer, prostate cancer, head and neck cancer, pancreatic cancer, and leukemia), cardiovascular diseases (coronary atherosclerotic heart disease, hypertension, and dilated cardiomyopathy), neurological diseases (stroke, Alzheimer’s disease, and dementia), chronic respiratory diseases (chronic obstructive pulmonary disease, and asthma), liver cirrhosis, chronic kidney diseases (chronic renal failure of all cause), and type 2 diabetes. Among the above-mentioned diseases, tumors are hotspots, followed by cardiovascular and neurological diseases. There were 15 articles (8.24%) in the literature describing TCM interventions aimed at reducing the incidence of NCDs, 167 (91.76%) articles describing the therapeutic effects on major diseases, 82 articles (45.05%) on malignancy, 35 articles (19.23%) on cardiovascular diseases, 29 articles (15.93%) on neurological diseases, 16 articles (8.79%) on chronic kidney diseases, 8 articles (4.39%) on liver cirrhosis, 8 articles (4.39%) on diabetes mellitus, and 4 articles (2.19%) on chronic respiratory diseases (Figure 3).

**FIGURE 4:**
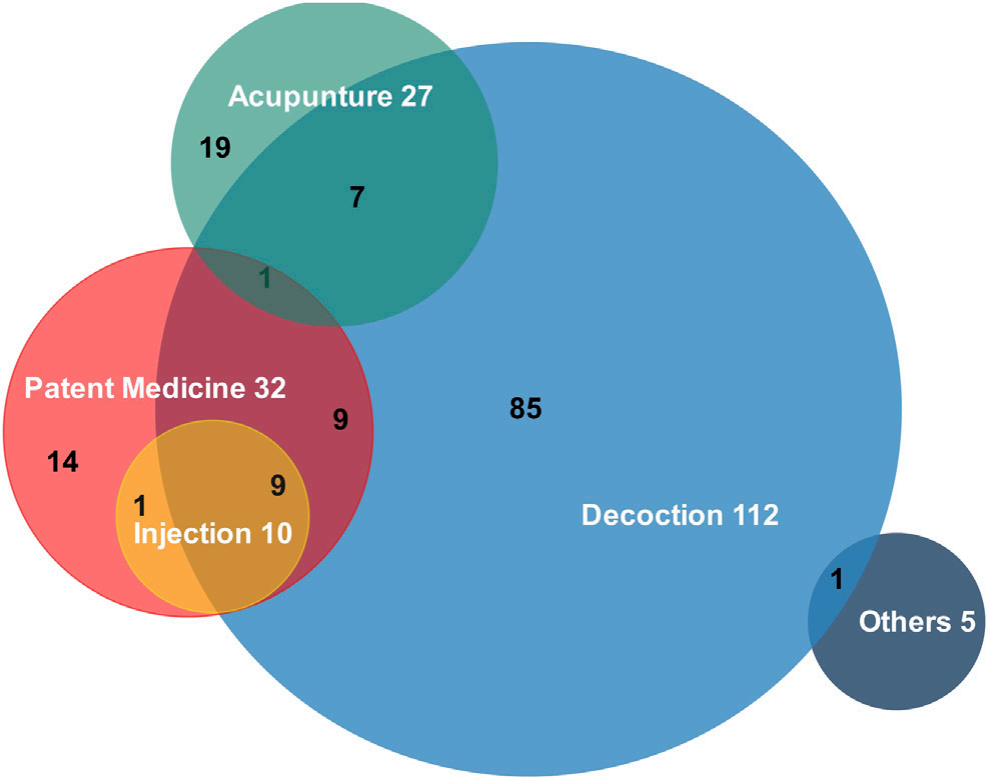
Wayne diagram for the exposure factors. Different exposure factors are shown as circles. Simultaneous modes of exposure are shown as intersections. Other modes of exposure include manipulation, TCM formula transdermal patches, TCM formula enemas, and TCM formula plasters. The white numbers are the total numbers of times of that exposure and the black numbers are the numbers of times of concomitant exposure.

The specific TCM interventions in the included articles were TCM decoctions, patent medicines (including effective extracts), TCM formula injections, acupuncture, and other interventions (manipulation, TCM formula transdermal patches, TCM formula enemas, and TCM formula plasters). Among them, 35 articles that did not specify the mode of exposure (without specifying the TCM intervention measure used), 85 articles used TCM decoctions, 32 articles used patent medicines, 10 articles used TCM injections, 27 articles used acupuncture as the mode of exposure, and 5 articles used other modes of exposure, with simultaneous exposure (Figure 4). There were 48 articles with an undefined duration of TCM exposure in the exposure group, none of which defined the cohort migration of patients and the corresponding statistical processing. Significant differences were noted in the selection of formulas and herbs in different studies on the same disease or in the same cohort; use of multiple combinations and selection of formulas make demonstrating the use of specific prescriptions difficult.

Owing to the varying characteristics of different diseases and the differences in the study objectives, a large number of observation indicators were involved, and some of the studies were exploratory and did not define the primary endpoints. There were 103 studies (56.59%) observing primary endpoint events (death, morbidity, etc.) and 65 studies (35.71%) observing secondary endpoint events (such as re-hospitalization rates and laboratory indicators). There were 14 studies (7.69%) observing only TCM syndrome evaluation, quality of life evaluation, or other subjective indicators.

The number of cohort studies on TCM for the prevention and treatment of malignant tumors is relatively large, and the selection of endpoint indicators is relatively uniform and representative. The main characteristics of the studies (published in English) are shown in Table 1. The main characteristics of another representative disease, the cardiovascular diseases, are shown in Supplementary Table S1.

**TABLE 1:** Overview of cohort studies on the treatment of malignant tumors with traditional Chinese medicine (published in English).

No	Diseases	Author	Year	Sample size	Exposure	Outcome	Follow-up time (months)	NOS score
1	Breast cancer	Huang ChingHui, et al. (Huang et al., 2019)	2018	48,914	Unspecified	Incidence of CHF (+)	36–204^a^	5
2	Breast cancer	Lee YiChiao, et al. (Lee et al., 2020)	2020	45	Chinese herbal decoction	OS (+); laboratory index (−); QOL (+)	40–52^a^	5
3	Breast cancer	Lee YuanWen, et al. (Lee et al., 2014)	2014	729	Unspecified	10 Year mortality (+)	12–56^a^	5
4	Breast cancer	Wang Yi, et al. (Wang et al., 2020a)	2020	148	Chinese herbal decoction	2 Year DFS (+); cumulative incidence rate (+); IDFS rate (+); AEs (−)	3–26^a^	7
5	Colorectal cancer	Yeh MingHsien, et al. (Yeh et al., 2020b)	2020	535	Chinese herbal medicine	Survival rates (+); subgroup analysis of survival rates (±)	36^∬^	4
6	Colorectal cancer	Shao cui, et al. (Shao et al., 2019)	2019	191	Chinese herbal decoction	OS (+); risk of death (+)	Unspecified	5
7	Colorectal cancer	Shi Qi, et al. (Shi et al., 2017)	2017	817	Chinese herbal decoction	DFS (+); subgroup analysis of DFS (±)	23–143^a^	6
8	Colorectal cancer	Xu Yun, et al. (Xu et al., 2017)	2017	312	Chinese herbal decoction; Chinese patent medicine	Recurrence and metastasis rate (+); OS (+); PFS (+)	60–82^a^	6
9	Colorectal cancer	Yang Yufei, et al. (Yang et al., 2008)	2008	222	Chinese herbal decoction Chinese patent medicine	Relapse and metastasis rates (+); time of relapse and metastasis (+)	12–60^a^	5
10	Colorectal cancer	Wang Yuli, et al. (Wang et al., 2020b)	2020	529	Chinese herbal decoction	Median PFS (−); subgroup analysis of median PFS (±)	12–72^a^	5
11	Colorectal cancer	Zhang Tong, et al. (Zhang et al., 2018)	2018	335	Chinese herbal decoction	Median OS (±)	11–39^a^	7
12	Gastric cancer	Hung KuoFeng, et al. (Hung et al., 2017)	2017	1924	Unspecified	OS (+)	12–170^a^	5
13	Gastric cancer	Shu Peng, et al. (Shu et al., 2019)	2019	489	Chinese herbal decoction	DFS (+); recurrence and metastasis rate (−); 5 year survival rate (+); QOL and TCM syndromes (±)	1–96^a^	6
14	Gynecological cancer	Zeng Yingchun, et al. (Zeng et al., 2018)	2018	30	Acupuncture	Neurocognitive Test performance (+); MRI and MRS (+)	Unspecified	5
15	Head and neck cancer	Lin HungChe, et al. (Lin et al., 2015)	2015	5,636	Chinese herbal decoction	Mortality rate (+)	1–132^a^	6
16	Leukemia	Tom Fleischer, et al. (Fleischer et al., 2017)	2017	498	Unspecified	Survival rate (+)	0–160^a^	5
17	Leukemia	Tom Fleischer, et al. (Fleischer et al., 2016)	2016	616	Chinese herbal decoction	HR of mortality (+); OS (+); most commonly prescribed TCM	28.68–34.2^∬^	3
18	Leukemia	Wang YuJun, et al. (Wang et al., 2016b)	2016	12,563	Chinese herbal decoction	OS (+); expenditure (−)	12–120^a^	5
19	Liver cancer	Liao YuehHsiang, et al. (Liao et al., 2015)	2015	127,237	Unspecified	OS (+)	24–144^a^	5
20	Liver cancer	Liao YuPei, et al. (Liao et al., 2020)	2020	14,729	Unspecified	HR of mortality (+); survival rates (+)	24–108^a^	5
21	Liver cancer	Sun Lingling, et al. (Sun et al., 2018)	2018	328	Chinese herbal decoction	Median OS (+); HR of mortality (+)	12–96^a^	4
22	Liver cancer	Zhang Wei, et al. (Zhang et al., 2014)	2014	191	Chinese patent medicine	Treatment effect (+); QOL (+)	16–19^a^	3
23	Lung cancer	Yeh MingHsien, et al. (Yeh et al., 2020a)	2020	1871	Chinese herbal decoction	Survival rate (+); mortality risk (+)	3–167^a^	4
24	Lung cancer	Shen HsuanShu, et al. (Shen and Wen, 2018)	2018	3,250	Unspecified	Lung cancer specific mortality (+)	Unspecified	4
25	Lung cancer	Li ChiaLing, et al. (Li et al., 2019)	2019	1988	Chinese herbal decoction	OS (+); PFS (+)	1–84^a^	5
26	Lung cancer	Liao YuehHsiang, et al. (Liao et al., 2017)	2017	111,564	Unspecified	Survival rate (+); risk factors and protective factors analysis	23.5–36.5^∬^	5
27	Lung cancer	Lin TsaiHui, et al. (Lin et al., 2019a)	2019	5,364	Unspecified	Incidence of lung cancer (+); risk factors and protective factors analysis	Unspecified	5
28	Lung cancer	Liu Jie, et al. (J. Liu et al., 2017)	2017	474	Chinese herbal decoction Chinese patent medicine herbal injection	OS (+); ORR (-); DCR (−); QOL (+); lung cancer-related symptoms (+); AEs (+)	Unspecified	7
29	Lung cancer	Wang XueQian, et al. (Wang et al., 2019)	2019	503	Chinese patent medicine Chinese herbal decoction	DFS (+); QOL (+)	0–40^a^	3
30	Lung cancer	Xiong ShaoQuan, et al. (Xiong et al., 2018)	2018	56	Chinese herbal decoction	DCR (+); median PFS (+); AEs (−)	12.3	5
31	Lung cancer	Zhao XueYu, et al. (Zhao et al., 2018)	2018	67	Chinese herbal decoction	Median OS (+); DFS (−)	7–66^a^	5
32	Lung cancer	Liu Rui, et al. (Liu et al., 2015)	2015	28	Chinese herbal decoction Chinese patent medicine herbal injection	Median PPS (+)	8–27^a^	5
33	Pancreatic cancer	Kuo YiTing, et al. (Kuo et al., 2018)	2017	772	Unspecified	HR of mortality (+)	12–180^a^	4
34	Pancreatic cancer	Yang Xue, et al. (Yang et al., 2015b)	2015	107	Chinese herbal decoction	Median OS (+)	1–57^a^	4
35	Prostate cancer	Lin PoHung, et al. (Lin et al., 2019b)	2019	248	Chinese herbal decoction	OS (+)	108–180^a^	4
36	Prostate cancer	Liu JuiMing, et al. (Liu et al., 2016)	2016	1,132	Unspecified	Survival rate (+)	1–96^a^	5
37	BPH/Prostate cancer	Kuo YuJui, et al. (Kuo et al., 2019)	2019	5,812	Chinese herbal decoction	Incidence of prostate cancer (+)	60–192^a^	5
38	Hepatitis B/Liver cancer	Tsai TzungYi, et al. (Tsai et al., 2017)	2017	21,020	Unspecified	Incidence of liver cancer (+)	0–180^a^	3
39	Colorectal cancer	Michael McCulloch, et al. (McCulloch et al., 2011)	2015	193	Chinese herbal decoction	Survival rate (+)	0–120^a^	4

Notes: AEs, Adverse Effects; APF, Alpha Fetoprotein; BPH, Benign Prostatic Hyperplasia; BMI, Body Mass Index; CHF, Chronic Heart Failure; COPD, Chronic Obstructive Pulmonary disease; DCR, disease Control Rate; DFS, Disease-free Survival; HR, Hazardous Ratio; IDFS, Invasive Disease-free Survival; KPS, Karnofsky Score; NOS, Newcastle-Ottawa Scale; OS, Overall Survival; PFS, Progression-free Survival; QOL, Quality of Life; RFS, Relapse Free Survival; SAS, Self-rating Anxiety Scale; SDS, Self-rating Depression Scale; TTP, Time to Tumor Progression.

^a^ Follow-up time is estimated from the time of enrollment to the time of the last follow-up (months). ∬Follow-up time is the mean follow-up time in the original article (months). (+) There is a statistically significant difference between the exposure and non-exposure groups; (−) there is no statistically significant difference between the exposure and non-exposure groups.

### NOS Scoring of the Articles

**FIGURE 5:**
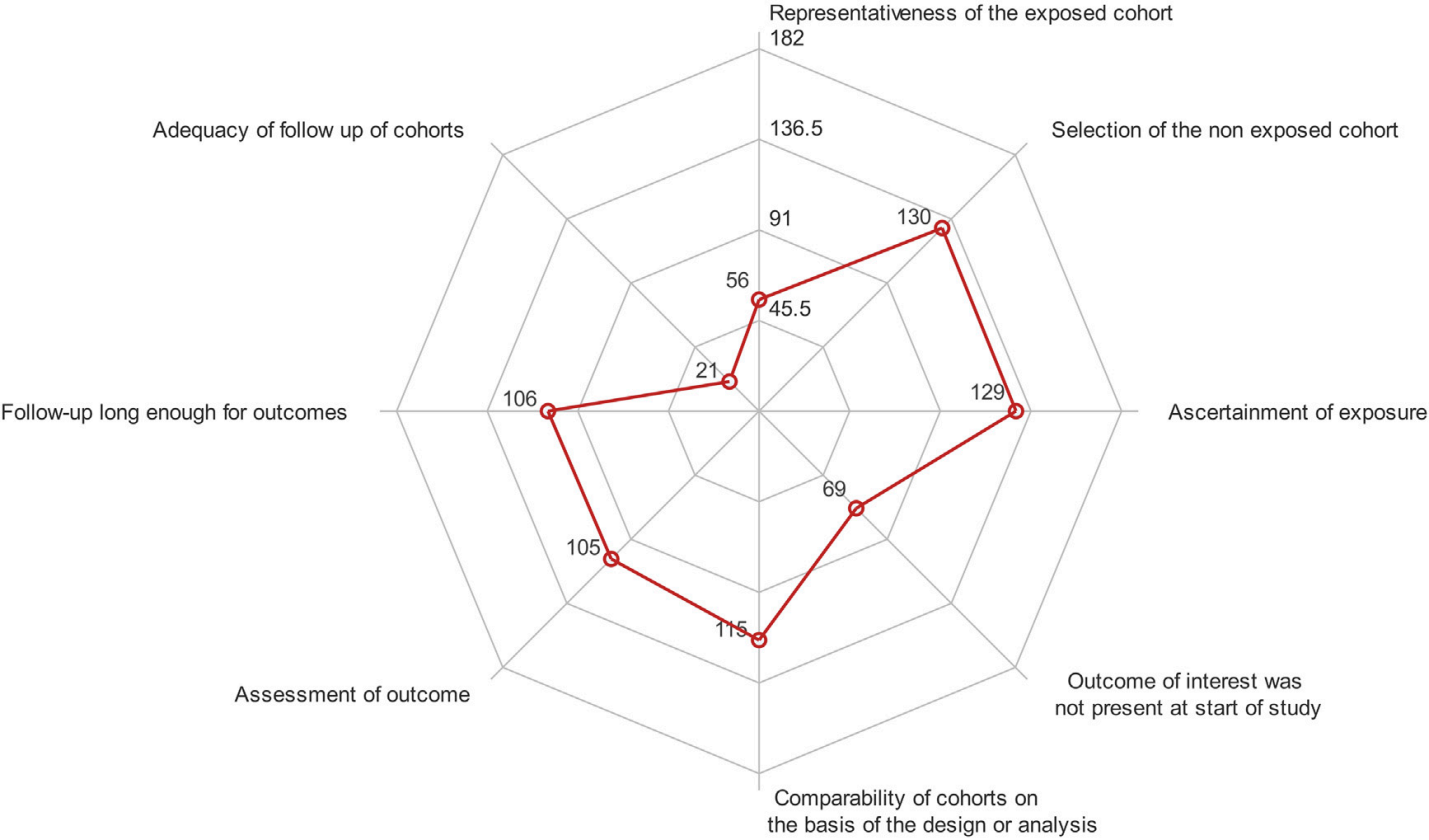
Radar plot of NOS scores of the articles. The eight rules of the NOS were used as quadrants, with the maximum value being the total number of articles (182). The “comparability” item was given a score of 0–2, and the remaining items were given a score of 0–1. The positions of red dots indicate the scores. The closer to the boundary, the higher the score. The area encircled by the red lines represents the quality of the studies as a whole. The larger the area, the higher the quality.

According to the eight rules of the NOS score, there were 11 articles (6.04%) with a NOS score of 7, 27 articles (14.84%) with a NOS score of 6, 67 articles (36.81%) with a NOS score of 5, 49 articles (26.92%) with a NOS score of 4, 22 articles (12.09%) with a NOS score of 3, and 6 articles (3.30%) with a NOS score of 2, thus showing that the methodological quality of most of the cohort studies was low (Figure 5).

### Status of Citations

**FIGURE 6:**
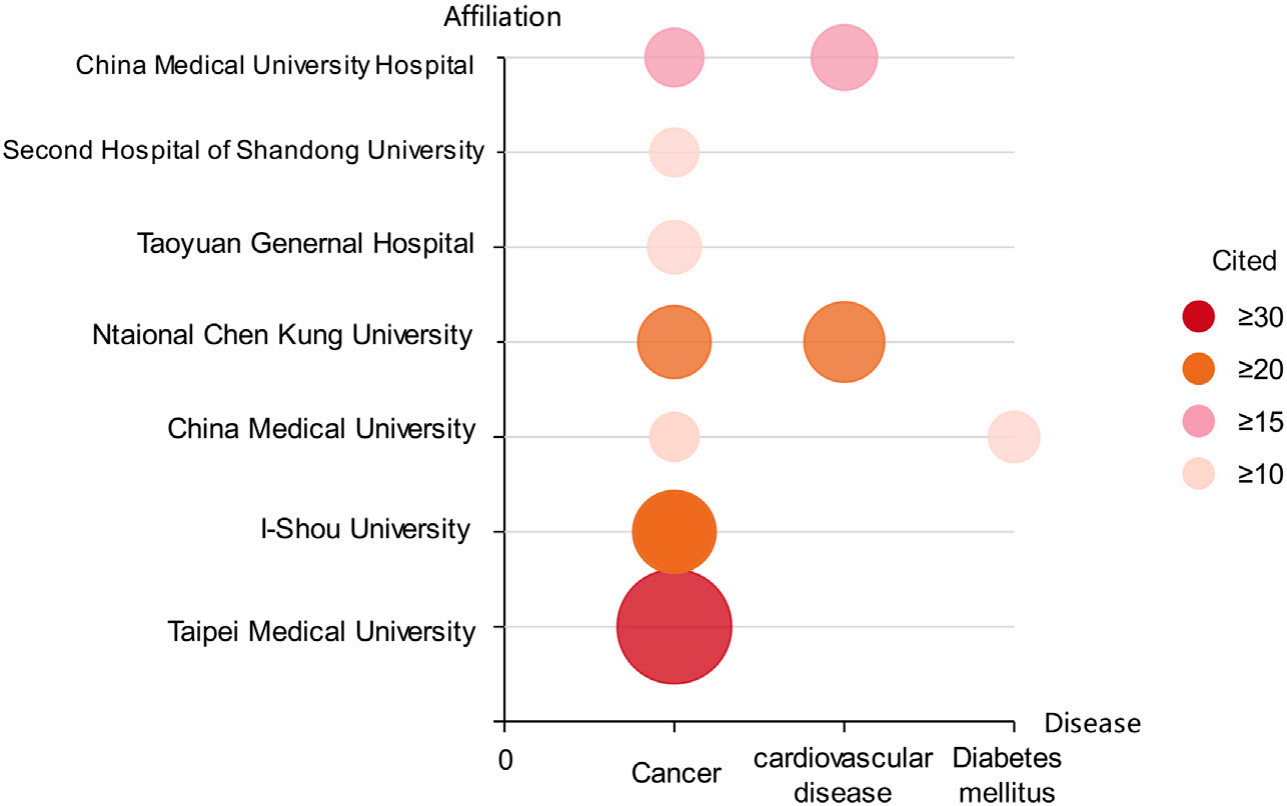
Schematic representation of citations. Articles with citations >10 as is shown in the figure. The size and color of bubbles represent the volume of citations.

The most frequently cited articles were breast cancer-related studies (citations = 38), and most of the other articles were cited less than 10 times. The average number of citations in the field of oncology was relatively high. The 13 articles (7.14%) with citations >10 were related to cancer, cardiovascular diseases, and diabetes mellitus, and most of the articles were published by affiliations in Chinese mainland and Taiwan (Figure 6).

## Discussion

Although cohort studies in the field of TCM were initiated relatively late in comparison to modern medicine (Nong et al., 2004), the number of such studies has rapidly increased and their quality has improved in the past decade. This study found that cohort studies on TCM for the prevention and treatment of NCDs had the following characteristics: 1) a cohort study on a malignant tumor is a hot and trending topic (45.05%), with a high number of articles and citations; 2) the number of studies in Taiwan has been growing rapidly, with large sample sizes; 3) some of the studies were poorly reported and did not clearly describe the type of TCM formula, dosage form, exposure time, and rates of loss to follow-up; 4) The quality of studies published in English was generally higher than that of studies published in Chinese, but high-quality cohort studies were still rare. The mortality rate of malignant tumors was found to be high. When conducting cohort studies, clinical endpoints such as mortality can be observed in a relatively short follow-up time, which is convenient for controlling the rate of loss to follow-up. Therefore, the number of cohort studies on malignant tumors was relatively high. Meanwhile, the quality of reports, impact factors of journals in which the articles were published, and citations of articles on TCM for cancer prevention and treatment were all higher than the average level of cohort studies in TCM. While oncology professional teams attached importance to and actively conducted cohort studies, the prevention and treatment of cancer with TCM have also received much attention. The large number of studies and included cases in Taiwan was because Taiwan has a well-developed national insurance system (Huang et al., 2015) that keeps records of medical consultations, diagnoses, and prescription information of patients covered by the medical insurance, which facilitates large-sample cohort studies. Thus, the improvement of the Hospital Information System and the nationwide networked health insurance system is conducive for the preservation of patient disease information, acquisition of a larger number of cases, reduction of selection bias when researchers are not blinded and patient recall bias, as well as the conduct of high-quality clinical studies, especially cohort studies.

Some studies were self-defined as cohort studies, but were actually randomized controlled trials (RCTs), case-control studies, or cross-sectional studies according to the reports (Yang et al., 2015a). The setting of exposure time varied widely among the studies, and there was no consensus or guideline on the exposure time of TCM, making it difficult to define whether the division between exposure and non-exposure was reasonable. The NOS scoring of all included articles revealed that articles with a score of four to five accounted for the largest proportion (63.73%). The following items were scored relatively low, which led to a decrease in the quality of evidence: representativeness of the exposed cohort and adequacy of follow-up of the cohorts. Most current studies recruit patients through outpatient or inpatient services, which will introduce a selective bias due to differences in the condition of the subjects and reduce the representative characteristics of the exposed cohort. Compared with studies from hospitals, cohort studies with community-based sources may be more representative of the population (AHRQ Methods for Effective Health Care, 2013). However, as an exposure, TCM needs to be prescribed by doctors, making it easier to recruit hospital cases in terms of obtaining a sufficient sample size and follow-up. None of the included articles describe a blinded follow-up, and most of them do not indicate loss to follow-up. A possible reason for this is that some researchers neglect following up retrospective cohorts or neglect normatively reporting patients lost to follow-up. However, most of the exposure groups in the studies had a lower rate of loss to follow-up than the non-exposure groups, suggesting that TCM formulas may be effective in clinical settings.

In addition to implementing and reporting studies according to the clinical research implementation guidelines (von Elm et al., 2008; Chinese Physicians Association of Integrative Medicine, 2015) and NOS (Stang, 2010), quality control for TCM cohort studies can be carried out in the following ways: clarifying the characteristics of the study methodology, as well as implementing and reporting the study appropriately. While cohort studies are suitable for diseases with relatively rare outcomes, case-control studies are more suitable for diseases with rare or unclear exposure factors and infectious diseases (Dupépé et al., 2019), and cross-sectional studies are used to describe the current status. The clinical registration and publication of study protocols in accordance with the clinical trial registration platform can reduce wastage of academic resources, increase the standardization of study implementation, and improve the credibility of studies. Reasonable follow-up methods and timely data entry of follow-up data should be ensured. The quality of follow-up visits can be improved to a certain extent by choosing a follow-up method with higher compliance and formulating a standard operating procedure for the follow-up.

With methodological advances, many high-quality cohort studies (Mahmood et al., 2014; Syddall et al., 2019) have provided new evidence for clinical practice. Cohort studies focusing on TCM still lack groundbreaking results, despite explosive growth in the number of published articles and significant improvement in the research quality (Wang et al., 2016a). While a large number of randomized controlled studies have confirmed that TCM can improve symptoms of diseases or certain laboratory parameters (Hershman et al., 2018; Song et al., 2019; Zhang et al., 2020), the long-term benefits should but not have been adequately evaluated in cohort studies. Compared to the recent development of high-quality RCTs on TCM, high-quality cohort studies are slightly lacking. Our literature retrieval revealed that the ratio of published TCM cohort studies to RCTs is much lower than the ratio of internationally published cohort studies to RCTs, indicating that there is room for development of cohort studies in real-world research. Although cohort studies have a lower level of evidence compared with RCTs (Djulbegovic and Guyatt, 2017), they allow individual subjects to select the treatment according to their willingness and facilitate personalized TCM treatment, improve the external validity of clinical studies, and provide evidence for the treatment of major diseases in real-world studies. Cohort studies are not a simplification of randomized controlled studies. These studies require a study design with a clear objective; cohort studies differ from RCTs in the difficulty and duration of follow-up rather than the implementation of the blinding. Clarification on whether a particular study is an exploratory or a validation study can achieve reasonable conclusions to some extent.

This study innovatively provides a bibliographic analysis of cohort studies on the treatment of chronic NCDs with TCM, which is rapidly developing in recent years, and summarizes research hotspots and methods to improve research quality, thereby providing a landscape for researchers interested in conducting cohort studies. However, we judged the methodological quality based on the reports in the retrieved articles, and the original authors were not contacted to obtain a research protocol or relevant items in the design; therefore, there may be instances where the actual research methods do not match those reported by the articles (Lo et al., 2014).

In summary, cohort studies on TCM for the prevention and treatment of NCDs have shown progress in recent years, but the overall quality is still not sufficiently high to provide a concrete basis for clinical decision-making. Some articles have pointed out that TCM for the prevention and treatment of NCDs can reduce the symptoms of diseases and the adverse effects caused by conventional treatment options, as well as improve the quality of life. Many basic studies have verified the effect of TCM on the pathological mechanisms of various NCDs from the aspect of inflammatory pathways, oxidative stress, and cell apoptosis (Teng et al., 2018; Tian et al., 2019; Chen et al., 2020). Therefore, in order to confirm the findings of these studies, cohort studies should strengthen the specific drug and acupoint composition of reporting the exposure factors (such as a specific formula and acupoint selection), provide more details on TCM, and should be conducted in combination with other basic experiments. In the future, reasonable control of bias and non-inferiority testing of mortality and other endpoints (Leung et al., 2020) to verify that there is no statistically significant difference, followed by reasonable scale scoring, can highlight the advantages of TCM in the prevention and treatment of NCDs. How to strengthen the quality control of studies and reduce bias, and how to make studies closer to the current clinical situation of TCM to provide more valuable evidence for the development of TCM are both issues that need to be further addressed.
