# Supplementary material for: Cohort Studies on Chronic Non-communicable Diseases Treated With Traditional Chinese Medicine: A Bibliometric Analysis
Source: Front Pharmacol. 2021 Mar 19;12:639860. doi: 10.3389/fphar.2021.639860 (PMC8017211; doi:10.3389/fphar.2021.639860)
Supplement: Supplementary file 1 [file table1.pdf]

Supplementary material Table S1. Overview of cohort studies on the treatment of cardiovascular diseases with Traditional Chinese medicine.

Number	Diseases	Author	Year/	Language	Area of implementation	Sample size	Exposure	Outcome	NOS score
1	coronary heart disease	Deng Peng.et al(1)	2018	Chinese	China(Jiangxi)	99	Other(hot compress)	ECG change(+)anginal frequency(+)TCM syndrome chart(+)adverse reaction(-)	4
2	arrhythmia/stroke	Chuang Sunfa.et al(2)	2015	English	China(Taiwan)	5180	Unspecified	morbidity of stroke(+)	5
3	coronary heart disease	Wu Huanlin.et al(3)	2012	Chinese	China(Guangdong)	537	Chinese Decoction	angina pectoris(+)ECG change(±)	5
4	coronary heart disease	Wu Huanlin.et al(4)	2009	Chinese	China(Guangdong)	49	Chinese Decoction Chinese Patent Medicine	readmission rate(+)angina pectoris(+)	2
5	coronary heart disease	Wu Huanlin.et al(5)	2009	Chinese	China(Guangdong)	296	Chinese Decoction	refractory anemia frequency(+) cardiac surgery(+)	2
6	coronary heart disease	Wang Xia.et al(6)	2013	Chinese	China(Guangdong)	190	Chinese Decoction	TCM syndrome(+) angina pectoris(+) life quality(+)	3
7	coronary heart disease	Wu Huanlin.et al(7)	2009	Chinese	China(Guangdong)	124	Chinese Decoction	refractory anemia frequency(+) readmission rate(+)	3
8	coronary heart disease	Yu Meili.et al(8)	2015	Chinese	China(Beijing)	220	Chinese medicine plaster	Nitroglycerin utilization(+) readmission rate in winter(+) TCM syndrome(+) AEs(-)	4
9	coronary heart disease	Gao Wulin.et al(9)	2018	Chinese	China(Shandong)	541	Chinese Decoction	composite end point of cardiovascular event(+)	2

							Chinese Patent Medicine Herbal Injection		
**10**	coronary heart disease	Gao Wulin.et al(10)	2018	Chinese	China(Shandong)	961	Chinese Decoction Chinese Patent Medicine Herbal Injection	cardiac mortality (±) composite end point of cardiovascular event (+)	5
**11**	coronary heart disease	Gao Wulin.et al(11)	2018	Chinese	China(Shandong)	707	Chinese Decoction Chinese Patent Medicine Herbal Injection	cardiac mortality (±) composite end point of cardiovascular event (+)	5
**12**	coronary heart disease	Yan Siyu.et al(12)	2019	Chinese	China(Beijing)	3004	Unspecified	composite end point of cardiovascular event(+)angina pectoris(-) TCM syndrome(+)	7
**13**	coronary heart disease	Zheng Wenguang.et al(13)	2010	Chinese	China(Shandong)	313	Chinese Decoction Chinese Patent Medicine	readmission rate(+) stroke(-)	6
**14**	coronary heart disease	Duan Wenhui.et al(14)	2012	English	China(Beijing)	334	Chinese Decoction	composite endpoint of cardiovascular events(+) readmission rate(-)	5

**15**	coronary heart disease & arrhythmia	Dai Guohua.et al(15)	2015	Chinese	China(Shandong)	313	Chinese Patent Medicine Chinese Decoction	readmission rate(+) mortality(-)	4
**16**	coronary heart disease arrhythmia	Huang Qi.et al(16)	2013	Chinese	China(Tianjin)	99	Chinese Patent Medicine	ECG change(+) TCM syndrome(+) readmission rate(-)	4
**17**	coronary heart disease& atherosclerosis	Du Wenting.et al(17)	2015	Chinese	China(Shanghai)	64	Chinese Decoction	cardiac function(+)BNP (+)echo cardiographic(+)Carotid ultrasound(+)serum lipid(+)AEs(-)	5
**18**	coronary heart disease(myocardial infarction)	Dai Guohua.et al(18)	2015	Chinese	China(Shandong)	238	Herbal Injection	Hospitalization: cardiac death(+) acute heart failure(-) cardiogenic shock(-) composite end point of cerebral infarction-stroke(-); Follow-up period: acute heart failure(+) cardiogenic shock(+) composite end point (+)	6
**19**	coronary heart disease(myocardial infarction)	Nong Yibing.et al(19)	2004	Chinese	China(Beijing)	162	Chinese Decoction	mortality(+) composite end point of cardiovascular event(+)	4
**20**	coronary heart disease/stroke	Chuang Sunfa.et al(20)	2017	English	China(Taiwan)	70695	Acupuncture	morbidity of stroke(+)	4
**21**	diabetes & coronary heart	Dai Guohua G.et al(21)	2018	English	China(Taiwan)	479	Chinese Patent	cardiac death(+) composite endpoint of	5

	disease						Medicine Herbal Injection	re-infarction and stroke(+)	
**22**	dilated cardiomyopathy	Wang Zhentao.et al(22)	2020	Chinese	China(Henan)	281	Chinese Decoction	echo cardiographic(+) dosage of metoprolol(+) dosage of urosemide digoxin(+) AEs(-)	4
**23**	dilated cardiomyopathy	Chai Songbo.et al(23)	2019	Chinese	China(Henan)	281	Chinese Decoction	echo cardiographic(+) Cardiac function(+)TCM syndrome(+)	3
**24**	heart failure	Ming-Yen Tsai.et al(24)	2017	English	China(Taiwan)	624	Unspecified	5-year survival rate(+)	6
**25**	heart failure	M. Habs (25)	2004	English	German	260	Chinese herb extracts	QOL(+) dosage of drugs(+) economic benefits(+)	4
**26**	hypertension	Jiang Fangchao.et al(26)	2017	Chinese	China(Shandong)	254	Chinese Decoction	cognitive function(+) blood pressure(+) life quality(+) TCM syndrome(+)	3
**27**	hypertension	Zhao Ming.et al(27)	2020	Chinese	China(Hebei)	151	Chinese Decoction	Symptom score(+) blood pressure(+) AEs(-)	5
**28**	hypertension	Cui Weifeng.et al(28)	2019	Chinese	China(Henan)	1364	Chinese Patent Medicine	AEs(+) composite end point of cardiovascular event(+)	5
**29**	hypertension	Ma Xiaofan.et al(29)	2019	Chinese	China(Henan)	5000	Chinese Patent Medicine	control of blood pressure(+)	3
**30**	hypertension	Cui Weifeng.et al(30)	2020	Chinese	China(Henan)	964	Chinese Patent Medicine	target organ damage (+)	3
**31**	hypertension	Chen Menglian.et al(31)	2018	Chinese	China(Zhejiang)	194	Chinese Decoction	blood pressure therapeutic effect(+)	4

							Acupuncture	high blood pressure control rate(+)	
**32**	hypertension	Chen KH.et al(32)	2017	English	China(Taiwan)	143382	Unspecified	morbidity of dementia(+)	4
**33**	hypertension	Hyejin Jung.et al(33)	2020	English	Korea	36022	Acupuncture	mortality(+)	7
**34**	hypertension	Youfu Ke.et al(34)	2013	English	China(Hongkong)	72	Chinese Decoction	heart rate(+) blood pressure(+) cardic function(+) AEs(-)	5
**35**	rheumatoid arthritis/coronary heart disease	Wu MY.et al(35)	2018	English	China(Taiwan)	19964	Acupuncture	morbidity of CHD(+)	4

Note: AEs(Adverse Effects), QOL (Quality of Life),ECG(Electrocardiograph), CHD(coronary heart disease), TCM(traditional Chinese medicine).

Reference

1. DENG P, HU D, WU JJ, XIONG J, XU Y, LIU ZY. Retrospective cohort study of 50 patients with chronic stable angina pectoris treated by traditional Chinese medicine hot election method. *Journal of Traditional Chinese Medicine* (2018) 59(05):398-401..
2. Chuang SF, Liao CC, Yeh CC, Lin JG, Lane HL, Tsai CC, et al. Reduced risk of stroke in patients with cardiac arrhythmia receiving traditional Chinese medicine: A nationwide matched retrospective cohort study. *Complement Ther Med* (2016) 25:34-8. Epub 2016/04/12. doi: 10.1016/j.ctim.2015.12.012. PubMed PMID: 27062945.
3. Wu HL, Xu DP, Luo WJ, Wang X. Clinical cohort study of anti-myocardial ischemia effect of Deng Tietao's scheme of regulating spleen and protecting heart in the treatment of coronary heart disease angina pectoris. *Liaoning Journal of Traditional Chinese Medicine* (2012) 39(03):385-7
4. Wu HL, Xu DP, Luo WJ. Retrospective cohort study of improving prognosis of patients after coronary artery bypass grafting by regulating spleen and protecting heart. *Jilin Traditional Chinese Medicine* (2009) 29(01):27-9
5. Wu HL, Luo WJ, Lin SS. A retrospective cohort study of 296 patients with coronary heart disease treated by regulating spleen and protecting heart. *Chinese Journal of Modern Medicine* (2009) 19(08):1220-2.
6. Wang X, Hu L, Li XQ, Wu HL. A prospective cohort study of regulating spleen and protecting heart in the treatment of coronary heart disease angina pectoris. *Journal of Guangzhou University of Chinese Medicine* (2013) 30(03):296-8+308.
7. Wu HL, Luo WJ, Huang YL. Retrospective study of regulating spleen and protecting heart in non-operative patients with stable angina pectoris. *Chinese Journal of Modern Medicine* (2009) 19(04):572-4..
8. Yu ML, Hu XM, Xu H. A prospective cohort study on the prevention and treatment of coronary heart disease angina pectoris with "winter disease and summer treatment" three-Fu patch. *Journal of Cardiovascular and Cerebrovascular Diseases of Integrated Traditional Chinese and Western Medicine* (2015) 13(09):1086-9.

9. Gao WL, Dai GH, Shi XJ, Zhao F, Yuan CH, Bi DX. Cohort study of TCM intervention in patients with coronary heart disease and unstable angina pectoris. *Chinese Journal of Experimental Formulae* (2018) 24(07):228-34.
10. GAO Wulin, DAI Guohua, WU Bin, GUAN Hui, SONG Chao, SUN Cong. Cohort study on intervention of traditional Chinese medicine in patients with myocardial infarction complicated with hypertension. *Lishizhen Medicine and Materia Medica Research* (2018) 29(09):2287-90.
11. Gao WL, Dai GH, Wu Bin, GH, Song C, Bi DX. Cohort study on intervention of traditional Chinese medicine in patients with myocardial infarction complicated with hyperlipidemia. *Chinese Journal of Traditional Chinese Medicine* (2018) 33(05):2002-6
12. YAN SY, LIANG XP, SU YN, GUO CX, LU PPi, LAN Y, et al.. Prospective clinical study of cardiovascular compound end points after PCI for coronary heart disease treated by traditional Chinese medicine. *Journal of Cardiovascular and Cerebrovascular Diseases of Integrated Traditional and Western Medicine* (2019) 17(17):2561-5.
13. Zheng WG, Dai GH, Han F, Zhao C. A cohort study of end point events in patients with coronary heart disease treated with traditional Chinese medicine during follow-up. *Hebei Traditional Chinese Medicine* (2015) 37(05):669-72+87.
14. Duan WH, Lu F, Li LZ, Wang CL, Liu JG, Yang QN, et al. Clinical efficacy of traditional chinese medicine on acute myocardial infarction: a prospective cohort study. *Chin J Integr Med* (2012) 18(11):807-12. Epub 2012/08/18. doi: 10.1007/s11655-012-1116-9. PubMed PMID: 22898760.
15. Dai GH, Zhao C, Zhang Y, Han F, Zheng WG. Cohort study on the application of Yin - boosting decrease internal heat calming prescription in 313 patients with coronary heart disease arrhythmia. *World Journal of Integrated Traditional Chinese and Western Medicine* (2015) 10(03):403-6.
16. Huang Q. Retrospective cohort study on the treatment of coronary heart disease with ventricular premature beats by nourishing blood and propitious wind. *Chinese Medicine Emergency* (2013) 22(08):1321-2.
17. Du WT, Liu Ping, Tang JY, Deng B, Tang N. Effects of the prescription of activating qi and activating blood circulation, promoting Yang and eliminating turbidities on patients with chronic heart failure and carotid artery plaque. *Chinese Journal of Integrated Traditional and Western Medicine* (2015) 35(11):1322-5.
18. Dai GH, Zhao C, Long FX, Lou H, Wang F. A cohort study of endpoints in 238 patients with myocardial infarction. *Journal of Traditional Chinese Medicine* (2015) 56(01):31-5.
19. Nong YB, Lin Q, Duan WH, Yang HL. A Cox proportional risk regression model for long-term prognostic factors of acute myocardial infarction from a retrospective cohort study. *Chinese Journal of Integrated Traditional and Western Medicine* (2004) (09):781-4.
20. Chuang SF, Shih CC, Yeh CC, Lane HL, Tsai CC, Chen TL, et al. Decreased risk of acute myocardial infarction in stroke patients receiving acupuncture treatment: a nationwide matched retrospective cohort study. *BMC Complement Altern Med* (2015) 15:318. Epub 2015/09/12. doi: 10.1186/s12906-015-0828-8. PubMed PMID: 26353964; PubMed Central PMCID: PMCPMC4563856.
21. Dai G, Gao W, Bi D, Liu C, Liu Y, Wang N, et al. Efficacy of Traditional Chinese Medicine in patients with acute myocardial infarction suffering from diabetes mellitus. *J Tradit Chin Med* (2018) 38(3):412-8. Epub 2018/06/01. PubMed PMID: 32185974.
22. Wang ZT, Liu SY, Chai SB, Gu ST, Bian RT. Cohort study of Kangqian Yixin prescription in the treatment of dilated cardiomyopathy. *Lishizhen Medicine and Materia Medica Research* (2020) 31(02):378-80.
23. Chai SB, Wang ZT, Liu SY. Cohort study of Kangqian Yixin Decoction in the treatment of Qi deficiency and blood stasis syndrome in dilated cardiomyopathy. *Shaanxi Traditional Chinese Medicine* (2019) 40(01):49-51.
24. Tsai MY, Hu WL, Chiang JH, Huang YC, Chen SY, Hung YC, et al. Improved medical expenditure and survival with integration of traditional Chinese medicine treatment in patients with heart failure: A nationwide population-based cohort study. *Oncotarget* (2017) 8(52):90465-76. Epub 2017/11/23. doi: 10.18632/oncotarget.20063. PubMed PMID: 29163845; PubMed Central PMCID: PMCPMC5685766.

25. Habs M. Prospective, comparative cohort studies and their contribution to the benefit assessments of therapeutic options: heart failure treatment with and without Hawthorn special extract WS 1442. *Forsch Komplementarmed Klass Naturheilkd* (2004) 11 Suppl 1:36-9. Epub 2004/09/09. doi: 10.1159/000080574. PubMed PMID: 15353901.
26. JIANG FC, ZHANG JY, XIE CX, ZHANG R. Kidney tonifying and Turbidity reducing prescription for the treatment of elderly hypertension with deficiency of Yin and Yang. *Jilin Traditional Chinese Medicine* (2017) 37(09):911-5.
27. Zhao M, Han NX, Yan YJ, Miao HW. A prospective cohort study of the treatment of Yin deficiency and Yang hyperactivity essential hypertension by Bushen Pinggan prescription. *Journal of Integrated Traditional Chinese and Western Medicine* (2020) 29(04):347-50+94.
28. Cui WF, Fan XH, Wang SF, Fan JX. A cohort study on the effect of long-term use of Chinese patent medicines to lower blood pressure on the outcome of patients with hypertension. *Chinese General Practice* (2019) 22(01):101-5.
29. Ma XF, Cui WF, Fan JM, Wang SF, Wang SR, Geng Y. The effect of Chinese patent medicines on blood pressure progression in patients with hypertension in the real world. *Liaoning Journal of Traditional Chinese Medicine* (2019) 46(10):2017-22+237.
30. Cui WF, Pan YY, Liang RF, Fan JM. Evaluation of the effect of traditional Chinese medicine series on the reduction of target organ damage in essential hypertension. *Chinese Journal of Gerontology* (2020) 40(11):2241-4.
31. Chen ML, Wei S. Effect of Traditional Chinese Medicine Comprehensive Intervention on Elderly Patients with Essential Hypertension in Community. *Medical Review* (2018) 37(S1):13-5.
32. Chen KH, Yeh MH, Livneh H, Chen BC, Lin IH, Lu MC, et al. Association of traditional Chinese medicine therapy and the risk of dementia in patients with hypertension: a nationwide population-based cohort study. *BMC Complement Altern Med* (2017) 17(1):178. Epub 2017/03/31. doi: 10.1186/s12906-017-1677-4. PubMed PMID: 28356117; PubMed Central PMCID: PMCPMC5372260.
33. Jung H, Yeo S, Lim S. Effects of acupuncture on cardiovascular risks in patients with hypertension: a Korean cohort study. *Acupunct Med* (2020):964528420920290. Epub 2020/06/23. doi: 10.1177/0964528420920290. PubMed PMID: 32567334.
34. Ke Y, Pu J, Zheng J. Essential hypertension treated by wuling powder and modified tianma gouteng decoction: a cohort study without controls. *Complement Ther Med* (2013) 21(6):609-12. Epub 2013/11/28. doi: 10.1016/j.ctim.2013.09.007. PubMed PMID: 24280468.
35. Wu MY, Huang MC, Liao HH, Chiang JH, Lee YC, Hsu CY, et al. Acupuncture decreased the risk of coronary heart disease in patients with rheumatoid arthritis in Taiwan: a Nationwide propensity score-matched study. *BMC Complement Altern Med* (2018) 18(1):341. Epub 2018/12/24. doi: 10.1186/s12906-018-2384-5. PubMed PMID: 30577824; PubMed Central PMCID: PMCPMC6303917.
